# Muscle building supplement use in Australian adolescent boys: relationships with body image, weight lifting, and sports engagement

**DOI:** 10.1186/s12887-020-1993-6

**Published:** 2020-02-26

**Authors:** Zali Yager, Siân McLean

**Affiliations:** 1 Institute for Health and Sport, Victoria University, Ballarat Road, Melbourne, 3011 Australia; 2 The Bouverie Centre, School of Psychology and Public Health, La Trobe University, Gardiner St, Melbourne, 3056 Australia

**Keywords:** Protein powder, Creatine, Anabolic steroids, Adolescent boys, Sports participation, Weightlifting, Body image, Prevalence, Predictors

## Abstract

**Background:**

The extent and implications of muscle building protein supplement use among adolescents is relatively unknown. This study aimed to describe the prevalence of protein powder, creatine, and anabolic steroid use in a sample of 14–16 year-old boys in Australia, and the predictors of actual use, and intentions to use protein powder.

**Methods:**

Data were obtained from questionnaires with Australian adolescent boys aged 14–16 years from one independent boy’s school in Melbourne (*N* = 237). Hierarchical linear and logistic regressions were used to determine the predictors of intentions, and actual use of protein powder.

**Results:**

49.8% of boys reported current use of, and 62% intended to use protein powder; 8.4% used creatine, and 4.2% used anabolic steroids. Higher levels of drive for muscularity, participation in weight training, and playing a greater number of sports were significant predictors of higher current use and intentions to use protein powder, but age, BMI, body esteem, and ethnicity were not.

**Conclusions:**

Prevalence of muscle building supplement use was relatively high among this adolescent population. This research has implications for intervention and prevention programs to educate young boys about muscle building supplements to reduce negative physical and psychological health effects of their use.

## Background

Over the past 10 years, Western countries have seen a rapid proliferation of the nutritional and muscle building supplement industries- which are now worth over $100 Billion USD, and increasing annually [1, 2]. The Asia-Pacific region, including Australia, has been identified as the area in which the fastest growth is expected, due to a focus on leading active and healthy lifestyles [3]. Muscle building supplements are widely available and yet they are poorly regulated [4], contributing to easy access, including by adolescents. These conditions contribute to high potential harm for adolescents.

Performance and Image Enhancing Drug [PIED], and muscle building supplement use among adolescent athlete and non-athlete populations is a concern for a number of reasons. First, there are potentially negative acute and chronic health effects of using supplements [5]. While natural whey protein may not cause harm, the impact of the use of creatine, and Anabolic Andronegic Steroids [AAS] among adolescents is relatively unknown [5, 6]. Research has also indicated that many muscle building products are spiked with testosterone or amphetamine-like substances, even though these are not declared on the label, which have relatively unknown effects on developing adolescent bodies and endocrine systems [7–9]. PIEDs use is also associated with multiple psychological harms, including body dysmorphia and depression [10]. There is also potential for a “gateway effect”, whereby the consumption of relatively benign substances can escalate and lead to more serious, and illegal supplement and substance use [11, 12]. For example, research has reported that competitive athletes were 3.5 times more likely to engage in doping if they had previously used nutritional supplements [12].

Prevalence of the use of muscle building supplements, including protein powder, creatine, and anabolic steroids, is relatively difficult to obtain with accuracy [13], but this information is important to ascertain, in order to raise awareness of the extent of harms associated with their use. Protein shakes and powders were reportedly used by 34.8% of high school adolescent boys in the US [14], and 25% of adolescent boys in Australia [15]. When studies are conducted among specific athlete groups, a higher prevalence of use is found, with 42% using whey protein powders, 29% using protein bars, and 29% using pre-mixed protein drinks [4]. The self-reported prevalence of creatine use among adolescents is around 5%, which is relatively low, considering that it is readily available [16]. Steroid use is much less common among adolescent boys, with prevalence reported to range from 1.5 to 5.9% in large studies of Australian and US high school males [14, 17, 18]. Large scale European prevalence data indicates that overall, 1% of adolescents have used anabolic steroids, but this was higher in some countries, including Bulgaria, where 7% of adolescent boys are using these substances [19]. In light of the expansion of the supplements industries and consequent increased availability of these products, understanding current prevalence rates in adolescents is important.

As well as understanding usage rates, identifying factors that contribute to use of supplements provides greater opportunities to intervene and prevent harmful use. Two broad factors have been identified as being linked to supplement use - a desire to enhance appearance, or a desire to enhance performance [20]. In addition to these factors, it is useful for researchers to fully understand the demographic, psychological, and contextual predictors of muscle building supplement use in order to more accurately prevent physical and psychological harm [21]. Gender is commonly accepted as a predictor of muscle building supplement use in that males are much more likely to use supplements and steroids than females [22, 23]. A relationship between ethnicity and the use of muscle building supplements seems to exist, but analyses of those relationships are limited to US populations [14].

In relation to appearance motivations for supplement use, research has found that adolescent boys who reported using muscle building supplements, including protein powders, were more likely to have higher levels of body dissatisfaction [24]. These findings are also replicated in US samples where drive for muscularity was found to predict changes in performance enhancing substance use and weightlifting behaviour [25]. In addition, common predictors of body dissatisfaction such as social appearance comparison, parent comments, and peer influences on appearance seemed to be particularly important in predicting the use of supplements in previous research [26]. Work with younger children (mean age ~ 9 years) also indicated that reductions in positive affect, and pressure to be muscular from the media, parents, and peers predicted the use of muscle-building strategies 16-months later [27].

Given the advertised effects of supplements on enhancing sporting performance, sports participation is likely to be a relevant contributing factor to PIEDs. Consistent with this contention, use of performance-enhancing substances is known to be more common among amateur and recreational athletes, for whom there are no penalties of supplementation [28, 29]. In relation to adolescents, research has found that those who are involved in sports teams were significantly more likely to use dietary supplements or to report use of protein powders for muscle enhancement than those who are not [14, 23]. However, the relationship between muscle building supplement use and participation in particular individual sports requires further research attention.

Given the broad access to supplements in Australia, it is important to understand the extent to which adolescent boys are using these substances, and the predictors of their use. This study aimed to explore the prevalence of protein supplement, creatine, and anabolic steroid use among Australian adolescent boys, and to determine the demographic, physical, and psychological factors that predict intentions and actual use of protein powder. It was hypothesised that after accounting for age, ethnicity, and BMI, lower body esteem and higher drive for muscularity would statistically predict greater intentions and actual use of protein powder. Furthermore, the addition of sports participation and weight training was expected to account for additional variance in both intentions and actual use of protein powder with greater sports participation and greater involvement in weight training being positively associated with intentions and with use.

## Methods

### Design

This research consists of secondary analysis of baseline data from the control and intervention groups (successive yearly cohorts) of an evaluation of the impact of a school-based steroid prevention program in adolescent boys (paper blinded for review) that was conducted in an all-boys school in Australia. High-risk human ethics approval was obtained from the (blinded for review) human ethics committee.

### Participants

Students in two successive cohorts of grade 10 (aged 14–16 years) at an independent boy’s school in Melbourne, Australia, were invited to participate in the research. Informed written consent was received from boys and their parents. A total of 237 participants, aged between 14 and 16, were included in the analysis.

### Procedure

Participants completed the online questionnaire, delivered via the survey software Qualtrics, in Health and Physical Education class time, on their school-issued devices, while supervised in ‘exam-like’ conditions by a researcher and their class teacher.

### Measures

The baseline questionnaire comprised standardized measures (see Table 1) and was used to collect data that would facilitate evaluation of the replication of the Athletes Learning and Training to Avoid Steroids [ATLAS] program. Therefore, the questionnaire included measures identical to those used in the original ATLAS evaluation research. Additional measures were used in the evaluation trial (Reference blinded for review) but are not reported here.

**Table 1:** Measures used to assess body image and supplement use

Variable	Measure
Demographics	Direct Questioning: Age, ethnicity, height, weight
Sports Participation	Boys were asked to indicate all of the sports that they are involved in. Options included: soccer, basketball, swimming, Australian Football, Cross Country, Hockey, Athletics, and ‘other (please specify)
Body Image	The 10-item Appearance subscale of the Body Esteem Scale for Adolescents and Adults [30] was used to assess body esteem. Cronbach’s alpha for the current study was acceptable α = .63
Drive for Muscularity	The 7-item Beliefs Subscale of the Drive for Muscularity Scale [31] was used to assess Drive for Muscularity. Previous research has confirmed that only the beliefs subscale, not the muscularity attitudes subscale predicts supplement use [25] Cronbach’s alpha for the current study was high α = .99
Use of Protein Powder, creatine, and anabolic steroids	Direct questioning items from ATLAS evaluation studies [32, 33] included: “Have you ever used (protein powder/creatine/anabolic steroids)” and participants responded by selecting an option from ‘I don’t know what this is’, ‘no, never’, ‘yes, once or twice’, or ‘yes, more than three times’.
Intentions to use protein powder, creatine, and anabolic steroids	Items from ATLAS evaluation studies [32, 33] asked participants to indicate the degree to which they agree or disagree (on a 7-point response scale) with the statements ‘I intend to try or use (protein powder/creatine/anabolic steroids)’.
Weight Training	Direct questioning “Do you do any weight training” with yes/no responses.

### Data analysis

All data was analysed using SPSS version 24. Two separate regression models were tested to assess for predictors of intention to use, and actual protein powder use among participants. Regression models focused on protein powder due to the low proportion of participants using either creatine or anabolic steroids. First, a hierarchical linear regression was conducted to determine predictors of *intention to use protein powder,* measured as a continuous variable using a 7-point response scale. Predictors entered into step one were demographic variables age, ethnicity, and BMI, followed by the addition of drive for muscularity (beliefs subscale) and body esteem (appearance subscale) at step two. Step three included two additional variables of ‘number of sports’ participants engaged in and weight training (yes/no; reference category = no). All categorical variables were dummy coded for entry into the models.

Second, a hierarchical logistic regression was conducted to assess predictors of *protein powder use* (measured using responses categorised as yes/no). Similar to the first analysis, predictors initially entered into the model included demographic variables of age, ethnicity, and BMI at step one, followed by drive for muscularity and body esteem at step two. The number of sports participants engaged in and weight training were added in step three of the model. Effect sizes were estimated using correlations and odds ratios.

Prior to regression analyses, the data was tested for assumptions of multicollinearity (Cut-out values: VIF=/> 10, Tolerance<.0.2), normality, homoscedasticity as well as outliers and influential cases. The assumptions were sufficiently met with only slight deviations from normality and homoscedasticity observed. Given the large sample size and the absence of multi-collinearity and influential cases, the data was considered robust to small irregularities and the analysis was continued.

To control for multiple comparisons, an adjusted significance value of α = .025 was applied. Due to the nested models within the analysis, a standard Bonferroni method was employed for the calculation of the adjustment. This method enabled the recognition of the two regression models as two hypotheses rather than treating each variable across two models as a separate test, and to reduce Type 1 error. A-priori power analysis for multiple regressions revealed that for an anticipated medium effect size (Cohen’s *f*^*2*^) of 0.15, a desired statistical power level of 0.80 and 7 predictors with a probability level of 0.01, the minimum required sample size is 147, so our sample of 237 exceeds this number.

## Results

### Demographics

Participants were asked to indicate their age from options of 13, 14, 15, 16, 17, or 18 years. The majority were aged 15 years (61.6%) and 16 years (36.3%). The mean BMI across the sample was 21.07 (*SD* = 3.47). The majority of participants were Caucasian (62.0%, *n* = 147), Southern European (16.5%, *n* = 39), Asian (8.9%, *n* = 21), ‘other’ (3.8%, *n* = 9), Indian or Sri Lankan (3.0%, *n* = 7), Aboriginal or Torres Strait Islander (2.1%, *n* = 5), Middle Eastern (2.1%, *n* = 5), or African (1.3%, *n* = 3).

### Sports participation

Participants’ self-reported engaging in a range of sports. The majority of participants listed participation in one sport (36.7%, *n* = 87), two sports (31.6%, *n* = 75), three (17.7%, *n* = 42), or four or more sports (13.1%, *n* = 31). Details of the type of sports that boys were engaged in are presented in Table 2. The sport with the highest proportion of participation was Australian Rules Football, followed by weight training and basketball. Participation in team and individual sports was relatively evenly represented.

**Table 2:** Proportion of participants engaged in different types of sports

Rank	Sport	Proportion %(n)
1	Australian Rules Football	45.6 (108)
2	Weight Training	41.8 (99)
3	Basketball	41.4 (98)
4	Other	21.5 (51)
5	Soccer	23.2 (55)
6	Swimming	16.0 (38)
7	Athletics	14.8 (35)
8	Hockey	14.3 (34)
9	Cross country	13.1 (31)
10	Cricket	12.2 (29)
11	Tennis	11.4 (27)
12	Martial arts/boxing	6.3 (16)

Most frequently reported sports in the “other” category were gym (3.0%, *n* = 7), rugby (2.5%, *n* = 6) badminton (2.5%, *n* = 6), and golf (2.1%, *n* = 5)

### Supplement use

Self-reported supplement use and intentions to use supplements are presented in Table 3. The most commonly reported supplement used was protein powder, with a little under half of the sample reporting use. Half of participants reported use of at least one of protein powder, anabolic steroids, or creatine supplements (50.2%, *n* = 119). The proportion of participants who intended to use supplements was higher than the proportion who had actually used supplements for each of protein powder, creatine, and steroids.

**Table 3:** Supplement use and intentions to use supplements

	% (*n*)		% (*n*)
Have used protein powder	49.8 (118)	Intend to use protein powder	62.0 (147)
Have used Anabolic steroids	4.2 (10)	Intend to use steroids	10.1 (24)
Have used Creatine	8.4 (20)	I intend to use supplements (eg. Amino acids, creatine)	25.7 (61)

### Predicting intentions and use of protein powder

Bivariate correlations (see Table 4) showed that muscularity beliefs, number of sports played, and engagement in weight training were all significantly associated with both intentions to use, and actual use of protein powder, such that higher muscularity beliefs and greater engagement in sports and weight training were associated with higher intention to use and greater use of protein powder. Effect sizes were small to moderate. With the exception of age, for which a small inverse relationship with intention to use protein powder was revealed, no other independent variables in the first model were significantly correlated with protein powder intentions or use. Older age was also associated with lower muscularity beliefs and greater body esteem of small effect size. As expected, body esteem had small inverse correlations with muscularity beliefs and with body mass index. Muscularity beliefs was positively correlated with weight training, of small magnitude, but not correlated with number of sports played. Number of sports played and weight training were positively correlated at small effect size. The positive correlation between protein powder intentions and use was of large magnitude.

**Table 4:** Correlations between demographic, body image, and sports engagement variables and intentions and use of protein powder

	Age	Ethnicity	BMI	DFM - beliefs	Body Esteem	Number of sports	Weight training	Protein powder - intentions	Protein powder - use
Age	–	−.011	.019	−.187**	.133*	−.025	−.107	−.127*	−.045
Ethnicity		–	−.122*	−.051	−.161**	.060	.041	.037	.012
BMI			–	−.039	−.209**	.033	.089	−.003	.002
DFM-beliefs				–	−.397***	.102	.221***	.304***	.207**
Body Esteem					–	−.055	−.049	−.044	−.024
Number of sports						–	.151*	.220***	.258***
Weight training							–	.481***	.357***
Protein powder - intentions								–	.648***
Protein powder - use									–

*Note*. DFM = drive for muscularity

*p < .05, **p < .01, ***p < .001

#### Predicting intentions to use protein powder

The summary statistics for the hierarchical linear regression analysis predicting intentions to use protein powder are presented in Table 5. At step one, age, ethnicity, and BMI accounted for 1.7% of the variance in intentions to use protein powder, which was not significant, *F* (3, 218) = 1.29, *p* = .280. After inclusion of body esteem (appearance subscale) and drive for muscularity (beliefs subscale) at step 2, the model accounted for 10.8% of variance, which was significant, *F* (2, 216) = 5.244, *p* < .001. After Bonferroni correction for multiple comparisons, only muscularity (beliefs) accounted for unique variance in intentions to use protein powder with greater muscularity beliefs predicting higher intentions. The final model in which number of sports played and weight training were added, significantly accounted for 29.8% of the variance in the intention to use protein powder, *F* (2, 214) =12.97, *p* < .001. Drive for muscularity (beliefs), the number of sports played and weight training each accounted for unique significant variance in intent. Higher drive for muscularity, participation in a higher number of sports, and greater engagement in weight training, significantly predicted greater intent to use protein powder, after controlling for age, BMI, and ethnicity. When also controlling for the effect of the number of sports, weight training remained the strongest contributor, accounting for 15.2% in the change in intentions.

**Table 5:** Summary statistics for hierarchical linear regression analyses determining statistical predictors of intention to use protein powder

	*R*^*2*^	*∆R*^*2*^	*∆F*	*B*	*SE*_*B*_	*β*	*p*
Step 1	.02	.02	1.29				
Age				−0.50	0.27	−.13	.061
BMI				0.002	0.04	.00	.958
Ethnicity				0.16	0.30	−.04	.595
Step 2	.10	.09	11.00***				
Age				−0.31	0.26	−.08	.235
BMI				0.02	0.04	.04	.580
Ethnicity				0.18	0.29	.04	.636
Muscularity (beliefs)				0.56	0.12	.33	< .001
Body esteem				0.03	0.02	.09	.176
Step 3	.30	.19	28.91***				
Age				−0.19	0.23	−.05	.416
BMI				−0.01	0.04	−.02	.746
Ethnicity				0.04	0.26	−.009	.884
Muscularity (beliefs)				0.37	0.11	.22	.001
Body esteem				0.02	0.02	.07	.279
Number of sports				0.21	0.09	.14	.019
Weight training				1.78	0.26	.41	< .001

*p < .05, **p < .01, ***p < .001

#### Predictors of current protein powder use

Summary statistics for the hierarchical logistic regression predicting current protein powder use are presented in Table 6. The first step of the model, containing age, ethnicity, and BMI was not significant, *X*^2^ [3] = 0.24, *p* = .972, and none of the independent variables significantly predicted protein powder use at this step. The inclusion of body esteem (appearance subscale) and drive for muscularity (beliefs) in the second step produced a non-significant model, *X*^2^ [5] =7.20, *p* = .206. Only muscularity beliefs significantly predicted protein powder use, with stronger beliefs predicting greater likelihood of using protein powder (*β* = .37, *p* = .001). For the final model, the inclusion of number of sports and weight training produced a statistically significant model, *X*^2^ [7] = 39.20, *p* < .001. Following adjustment for multiple comparisons (critical alpha cut-off set at .025), only number of sports played and weight training contributed significantly to the model. The strongest predictor of use of protein powder was weight training. Boys who trained with weights were 3.8 times more likely to report use of protein powder than boys who did not. In addition, for every additional number of sports played, boys were 1.4 times more likely to use protein powders.

**Table 6:** Summary statistics for hierarchical logistic regression determining statistical predictors of current protein powder use

	*X*^2^ *(df)*	*∆ X*^2^ *(df)*	*B*	*SE*_*B*_	Wald	*df*	*p*	Odds Ratio	95% CI for Odds Ratio
Step 1	0.24 (3)	0.24 (3)							
Age			−0.08	.268	0.08	1	.773	.927	(.56, 1.55)
Ethnicity			0.02	0.28	0.01	1	.944	.980	(.59, 1.78)
BMI			0.02	0.04	0.15	1	.694	1.02	(.94, 1.10)
Step 2	7.20 (5)	6.96 (2)*							
Age			0.00	.27	0.00	1	.999	1.00	(.59, 1.69)
Ethnicity			−0.06	.30	.0.04	1	.846	.945	(.54, 1.67)
BMI			0.03	0.04	0.38	1	.540	1.03	(.95, 1.76)
Muscularity (beliefs)			0.32	0.13	6.43	1	.011	1.37	(1.08, 1.76)
Body esteem			0.01	0.02	0.316	1	.574	1.01	(.97, 1.06)
Step 3	39.20 (7)***	32.01 (2)***							
Age			0.01	.29	0.00	1	.969	1.15	(.57, 1.80)
Ethnicity			0.08	0.31	0.06	1	.810	1.08	(.58, 2.00)
BMI			−0.00	0.05	0.00	1	.963	1.00	(.91, 1.09)
Muscularity (beliefs)			0.21	0.14	2.45	1	.117	1.24	(.95, 1.62)
Body esteem			0.007	0.02	0.09	1	.763	1.00	(.96, 1.05)
Number of sports			0.36	0.13	8.55	1	.003	1.44	(1.13, 1.84)
Weight training			−1.35	0.31	19.09	1	< .001	3.84	(2.10, .7.02)

*p < .05, **p < .01, ***p < .001

## Discussion

In this paper, the prevalence and predictors of the use, and intentions to use protein powder were reported among Australian adolescent boys. Half of the participants had used protein powder, and 62% intended to use protein powder. Use of creatine and anabolic steroids was lower than use of protein powder, but greater proportions of participants reported intentions to use these products. The number of sports that boys participated in, and whether or not they engaged in weightlifting, were the strongest statistical predictors of use, and intentions to use protein powder- more so than body esteem, drive for muscularity, age, ethnicity, or BMI. Our hypotheses were partially supported, in that sports participation and weight training, as well as drive for muscularity, were significant predictors of use, and intentions to use protein powder. In contrast to our hypotheses, body esteem was not a predictor of the use, or intentions to use protein powder.

The prevalence of the use of protein powder in this universal school sample is much higher than prevalence from data collected among a US sample 5 years ago. In the Project Eat study, 34.7% of adolescent boys (mean age 14 years) were using protein shakes and 5.9% reported using steroids [34]. The prevalence found in our study was more closely aligned with an Australian sample from a similar time period, that found that 42% of adolescent athletes (13–18 years) had used whey protein powders, 29% consumed protein bars, and 29% used pre-mixed protein drinks [4]. Replication with other universal samples will be required to determine whether supplement use is higher among Australian adolescent boys than adolescent boys from the US or elsewhere, or whether usage rates are increasing over time.

The high use of muscle building supplements found in the current study is concerning due to their potential physical [5] and psychological [20] negative health effects. The consumption of protein powders is dangerous due to the potential for adolescents to consume unknown substances, with which protein products may have been “spiked” [8, 9]. In addition, there is the potential for a “gate-way effect”, in that the use of socially acceptable products like protein powders might lead to use of more harmful and illegal substances [11, 12]. The fact that these protein powders are readily available, and frequently used [24], compounds these concerns.

In this study, sports participation and weight training were associated with greater use, and intentions to use protein powder. This is consistent with past research with adolescent boys- which has also generally found that those who are playing sports are more likely to use muscle building substances. However, the exact nature of the association is not yet clear. In the Project Eat study, adolescents participating in sports were significantly more likely to report muscle-enhancing behaviours, including the use of protein powder [14]. In a systematic review of 52 studies investigating the factors predicting the use of doping and performance enhancing substances in young people, Nicholls and colleagues concluded that the relationship between sports participation and doping use was unclear. Of the five studies that were reviewed, some found no differences among athletes and non-athletes, higher use among athletes, or higher use among non-athletes [22]. The review did, however, demonstrate that consistent with our findings, there was a clear relationship between engagement in strength-based sports and activities, for example weight training, American wrestling, and American football, and PIED use [22].

The current study findings are consistent with past literature that has reported greater supplement use in adolescents with body dissatisfaction [24]. The present results add greater specificity to past literature by demonstrating that muscularity beliefs, but not body esteem, were associated with higher intention to use, and actual use of protein powders. This distinction is important, as it demonstrates that it is the specific focus on concerns about muscularity, rather than general perceptions about appearance that are related to engagement in body change strategies in adolescent boys. This information provides a useful insight into potential targets for intervention, suggesting that improving body image in general, rather than focusing on muscularity specifically, would be likely to be less effective to address supplement use.

The supplements and doping literature is filled with examples of limitations to research due to the nature of the questions and scales used, and this research is no different. Given the limited attention span of adolescent boys, it is important that questionnaires are kept as short as possible, but this means that layers of detail are missed. In this study, direct questions were used to determine the use, and intentions to use protein powder, but it is possible that there are better means of determining this information. Questions about sport participation could also have been more specific, in order to determine the level (amateur, recreational, elite, etc) and amount of the sport played (occasionally, one per week, etc). More objective physiological measures such as accelerometers could have provided a more accurate reflection of physical activity engagement.

### Implications for research and practice

This research found high prevalence of use of protein products in mid-adolescent boys, and this has implications for the development and implementation of education, prevention, and intervention programs in a range of settings. First, this research justifies the inclusion of education about PIEDs and supplements in the school curriculum, aimed at a universal audience. In terms of timing, findings of relatively high protein powder use among 14–16 year-old adolescents suggests that it is important that intervention and education programs to prevent supplement use take place when boys are 12–13 years old, before these attitudes and behaviours are established.

This research found that adolescents who were engaged in weight lifting were particularly more likely (3.8 times) to be engaging in current protein powder use. This presents an opportunity to engage a potentially high-risk group of boys in selective intervention program to prevent current harm, and future PIEDs use. Education and professional development programs for teachers, personal trainers, gym and fitness centre staff, and coaches is also required in order to support programs targeted at boys. Whole-of-centre approaches towards making gyms, fitness centres, and other settings where weight lifting occurs more supportive of health (as opposed to appearance-related) motivations for physical activity are also advised.

In terms of the content of education programs, it is suggested that, as a field, we look towards other evidence-based drug education programs that have been found to prevent the use of drugs and alcohol use among adolescents. Some school-based drug education programs designed for universal groups, regardless of their risk level, have produced small to moderate effects in terms of health behaviour change [35, 36]. Specific doping prevention interventions have also been effective, for example the Hercules program [37], and the Athletes Training and Learning to avoid Steroids [ATLAS] program [38], indicating potential directions for prevention programming.

## Conclusion

Almost one in two adolescent boys aged 14–16 years reported using protein powder in this study. Use of protein supplements was linked to participation in weight lifting, the number of sports that the boys were engaged, and drive for muscularity- although the sports related factors were more important in predicting intention and use than the body image or demographic factors. This research provides important prevalence data that can inform clinical paediatric practice, as well as intervention and prevention programs.

Paediatricians should be aware of the high proportion of adolescent boys using protein powders and other muscle building supplements. Questioning patients about their weight training and muscle building supplement use is appropriate in order to determine whether boys are using supplements that might affect mood, growth, endocrine, and cardiovascular functioning. Advising boys who are currently lifting weights against using supplements with high levels of caffeine, and other ingredients is warranted.

There is very little attention to supplement education in the Australian Health and Physical Education Curriculum [39], and when supplement use is raised, in the optional Victorian Certificate of Education [VCE] Health and Physical Education syllabus, the focus is on doping in sport, and not a critique of the wider use of performance and image enhancing substances. Parents, teachers, and adolescents, should be informed about the potential impact of using these supplements, and specific indicated interventions for boys who are engaging in weight-lifting should be developed and trialled.

## Data Availability

Datasets used and/or analysed during the current study are available from the corresponding author on reasonable request by email.

## Abbreviations

AAS: Anabolic adrenergic steroids
ATLAS: Athletes training and learning to avoid steroids program
BMI: Body mass index
DFM: Drive for muscularity
PIEDs: Performance and image-enhancing drugs
VCE: Victorian certificate of education
